# Improving national-scale breeding bird surveys with integrated distance sampling

**DOI:** 10.1038/s41598-025-96787-w

**Published:** 2025-05-26

**Authors:** Jean Nabias, Romain Lorrillière, Jérémy Dupuy, Laurent Couzi, Luc Barbaro

**Affiliations:** 1 LPO-BirdLife France, Fonderies Royales, Rochefort Cedex, France; 2 CESCO, Muséum National d’Histoire Naturelle, CNRS, Sorbonne-University, Paris, France; 3 Centre de Recherches sur la Biologie des Populations d’Oiseaux (CRBPO), MNHN-CNRS-OFB, Paris, France; 4 Dynafor, INRA-INPT, University of Toulouse, Auzeville, France

**Keywords:** Bird monitoring, Citizen science, Distance sampling, Data integration, Hierarchical modelling, Observation process, Ecological modelling, Statistics

## Abstract

Bird population estimation over broad spatial and temporal scales is a key objective in ornithology. To date, bird ecologists mainly relied on standard point counts where the number of detected individuals is interpreted as either the true abundance or proportionally related to it. However, providing accurate estimates of species abundance requires modelling the observation process with temporally replicated data, which is not always possible with the increasing use of ever-bigger datasets from citizen science programs. Data integration methods allow combining temporally replicated sampling at coarser spatial grains with data collected over larger spatial extents. Here, we developed an Integrated distance sampling (IDS) to combine national structured and semi-structured citizen-based bird surveys in France to estimate species abundances using observation distances and accounting for availability, i.e. the probability of individuals being detectable during a given sampling visit. While our simulation study showed an overall increase in the accuracy of estimated parameters for both ecological and observation processes, without significant biases, our case study suggests that such model improvements will depend on specific sampling scenarios. Integrated models represent a promising tool for ecological science, permitting the joint use of large unstructured datasets with scale-restricted structured surveys.

## Introduction

To estimate bird species abundance, ornithologists mainly rely upon standardised point-count methods consisting of individual records of detected birds, either visual or auditory, over a given time period^1^. While standard models like GLMs (generalised linear models) allow extrapolating these observed counts to novel unsampled conditions through covariates, they also imply that the number of detected individuals represent an accurate estimate of true abundance, or corresponds to a constant proportion of the sampled population across space and time^2,3^. However, multiple studies have shown that this assumption is not always viable^2,4,5^ because of variations in species detectability arising from observation errors^3^, or changes in species phenologies^6^. can affect the actual proportion of detected individuals.

For a set sampling effort, data collection faces a trade-off between (i) the sampling of a large quantity of unstructured data across a broad spatial scale, or (ii) sampling of highly standardised data collected at a smaller scale^7^. Given the nature and volume of data collected by standard protocols, ecologists must address this issue relying increasingly on more or less opportunistic or semi-structured Citizen Science (CS) programs^8^. However, reliable abundance estimates require additional information such as repeated visits, collection of detection distances or data collected by multiple observers, to enable the combined modelling of the distinct ecological and observation processes^9^, see Box 1.

While the ecological process corresponds to species response to environmental covariates variations through space and/or time, the observation process depicts a probabilistic representation of mechanism underlying data collection^10^. Nichols et al.,^11^ describe the observation process as being represented by four components; (i) the probability that individuals’ home ranges overlap the sampling units ${p}_{s}$; (ii) given ${p}_{s}$, the probability that individuals are present on the sampling units during observers visits ${p}_{p}$; (iii) the probability that individuals’ are available for detection (for instance, bird vocalizing during observer visits) denoted ${p}_{a}$ and (iv) the probability of detection given individuals presence and detectability ${p}_{d}$. While ${p}_{s}$ is assessed through sampling design and ${p}_{d}$ can be inferred from specific data collection, such as detection distances; ${p}_{a}$ and ${p}_{p}$ probabilities require temporal replicates to be estimated^12^.

Ecological inferences, explicitly accounting for the ecological and observation processes, require flexible statistical tools such as hierarchical models able to account for global model complexity by a succession of submodels of lesser complexity^13^. These models vary depending on the studied ecological process^14^ from species presence/absence – (occupancy models; Ref.^15^) to species abundance – (hierarchical distance sampling,^16^; N-mixture models,^17^) or demographic parameters estimation – (Cormack-Jolly-Seber models, Ref.^18^).

In the last two decades, Citizen Science has seen exponential growth^19^ thanks to the development of several online databases such as eBird (www.ebird.org), iNaturalist (www.inaturalist.org) and GBIF (www.gbif.org) aiming to handle observation data collected by volunteers^20^ over increasingly longer temporal and larger spatial scales^21^. These databases rely mostly on opportunistic data, information gathered without sampling design or focused taxa^21^. While the use of metadata and ad hoc filters can increase the value of collected data^22^, Citizen Science tends to lack specificities of structured surveys, including intra- and inter-year repeated visits^23,24^.

Data integration, or the simultaneous joint analysis of an ecological process using multiple datasets^25^ developed a growing interest in recent years^26,27^. It is used, for instance, in the case of complex ecological inference requiring different data sources, such as integrated population models (IPM;^25^). These models rely on count data as well as nest monitoring and/or banding to infer population spatiotemporal variations and population growth parameters^28,29^; or to combine data collected at different spatial and/or temporal resolutions^30^.

Here, we focus on data collected for breeding bird atlases, depicting known distribution and population size estimates using data collected over a short timeframe. In France, the previous breeding bird atlas^31^ was based on a semi-quantitative method to estimate national population size^32^. This approach extrapolated bird densities locally determined over a few local areas without accounting for the detection process. It resulted in biased estimations of French breeding bird populations when compared to estimates inferred from a structured CS scheme EPOC-ODF (Structured Estimation of Common Bird Population Size, see^33^). While structured schemes result in intensive data collection to collect high-quality data, they tend to be conducted over a rather limited spatial extent. In contrast, semi-structured schemes aim at overcoming this issue to gather interpretable data while still enlisting the largest possible number of observers and associated field data^34^. For our study, we used datasets from both the structured CS scheme EPOC-ODF and the semi-structured CS scheme EPOC (Estimation of Common Bird Population Size), where one scheme allows inference of the detection process through repeated visits, while the other focuses on the collection of environmental data without repeated visits, akin to a double-sampling design^35^.

Recent studies have shown the potential of data integration on ecological inferences combining data from multiple data sources for occupancy modelling^36,37^ and species abundance estimates^38^. In this study, we relied on a joint likelihood approach^39^ based on the integrated distance sampling (IDS) formulation from^38^. While Kéry et al.,^38^ formulated an IDS model integrating data from unreplicated distance sampling data using point count and detection/non-detection data assessing species availability through list duration, we aim to calibrate an IDS model accounting for species availability through temporal replicates. Availability, or temporary emigration^11,12,17^, can represent different biological processes, such as (i) random temporary emigration, when individuals display conspicuous behaviours allowing increased detection rate during survey (birds vocalisations^40^, burrowing or diving^41,42^); (ii) spatial temporary emigration, where individuals remain undetected due to being physically outside the sampled sites during survey period; and (iii) availability resulting from variation in population-level processes, such as recruitment, survival, emigration or immigration^13,43^. Survey duration, addressed in^38^, accounts primarily for random temporary emigration where individuals could be present on site but remained undetected due to a lack of emitted vocal or visual cues. In contrast, temporal replicates across broader time scales, used in this study, mainly account for spatial temporary emigration instead.

In this manuscript, we applied the developed IDS model to a structured and semi-structured dataset, EPOC-ODF and EPOC, collected over three French regions under distinct data collection schemes. We compared ecological and observation parameters estimates from the IDS model to those obtained from a HDS model calibrated using only data collected by EPOC-ODF to test if data integration could lead to improvement in the accuracy of estimated parameters, *i.e.* reduction of their uncertainties. In addition, we conducted a simulation study aiming (i) to assess model identifiability, *i.e.*, its capabilities to accurately estimate parameters; and (ii) to test potential improvement in estimated accuracy over multiple ranges of variation of simulated species availability, detectability and sampling scenarios.

## Material and methods

Hierarchical distance sampling Hierarchical distance sampling (HDS) model aimed to estimate species abundance while taking account of the observation process^13^. As conventional distance sampling assumes perfect detection^44^ at a null distance from the observers (i.e. $f(x=0) = 1$, see below), HDS can relax this assumption by assessing the probability that the individual is present and available for detection during survey occasions^17^ through lists duration or multiple visits at the same site. Considering a population following Poisson distribution with mean ${\lambda }_{i}$, at each site i = 1,2,..,I we have the local population size ${M}_{i}$:

$${M}_{i}\sim Poisson({\lambda }_{i})$$

Given multiple visits *j* (*j* = 1,2, ... ,J), at site *i*, the number of individuals available for detection ${N}_{i,j}$ follows a binomial distribution from the local population ${M}_{i}$ with a probability of being exposed to sampling, i.e. available for detection, ${\varphi }_{i,j}$:

$${N}_{i,j}\sim Binomial({M}_{i},{\varphi }_{i,j})$$

For each site *i* and visit *j*, observers measure the distance of observation between themselves and detected individuals. A vector of cell probabilities ${\pi }_{i,j}$ derived from a detection function *f*^44^, assigns probabilities to distinct distance bins. Observation ${y}_{i,j}$ can then be described as a multinomial outcome given the number of individuals available for detection and its distance (${x}_{i,j}$):

$${y}_{i,j}\sim Multinomial\left({N}_{i,j},{\pi }_{i,j}\right), with\ {\pi }_{i,j}=f({x}_{i,j},\sigma )$$

In our study, we relied on point count data using observation distances between observers and detected individuals. We also considered a half-normal model, with parameter ($\sigma$) for the detection function.

### Simulation study 1: model identifiability

**Fig. 1:**
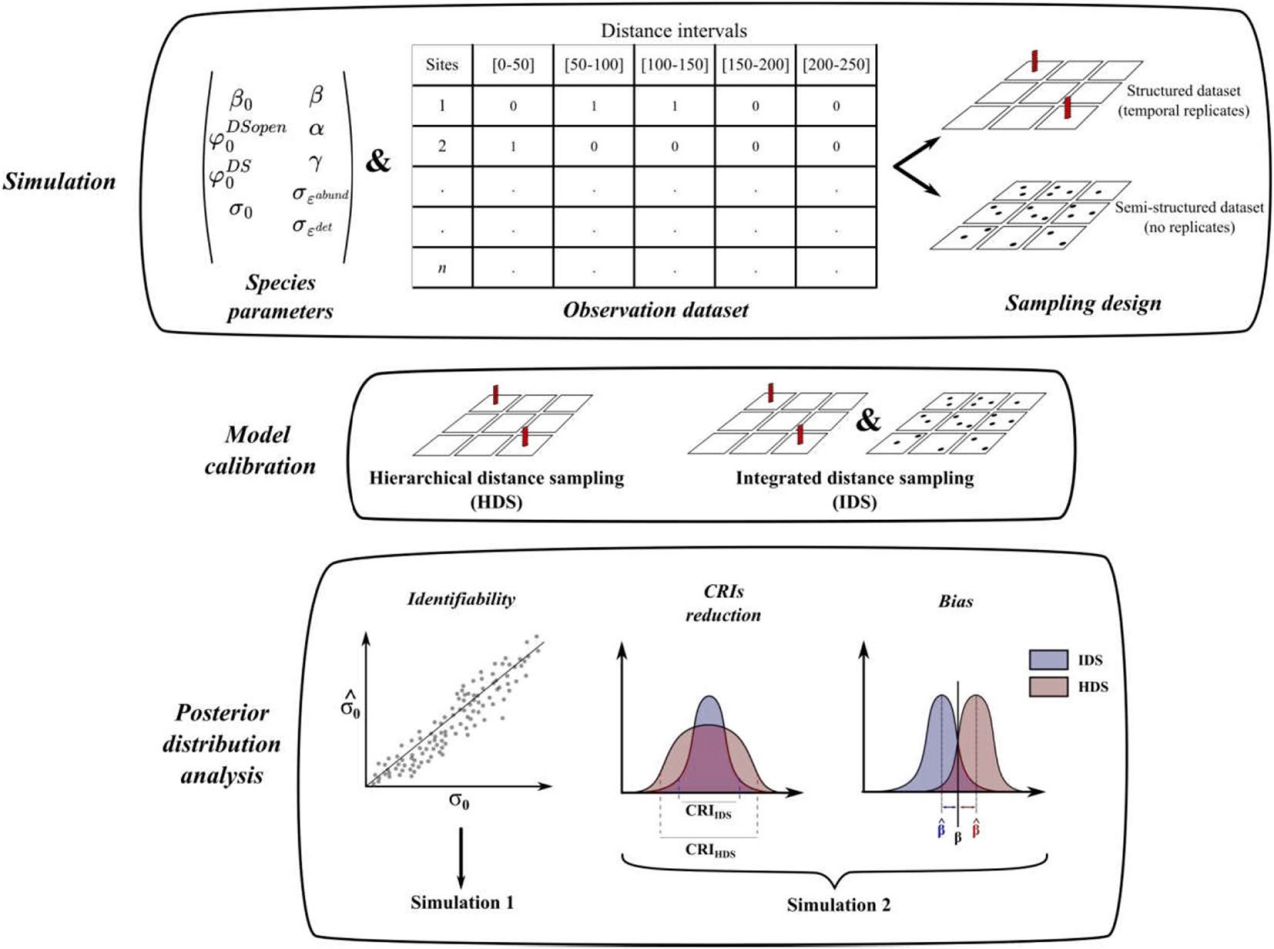
Schematic representation of the simulation study design. We simulated 1000 datasets (structured and semi-structured) using the same set of simulated parameters across the two simulation studies. We simulated distinct detection probabilities ${\varphi }_{0}^{DSopen}$ and ${\varphi }_{0}^{DS}$ for each sampling design aiming to mimic a protocol effect.

For simulation study 1, we generated 1000 cases each consisting of a structured dataset, with 9 temporal replicates, collected over 200 sites and a semi-structured dataset containing 1000 sites with single visits over one season (Fig. 1). For each case, we randomly generated sets of parameters related to the ecological and observation processes, with (${\beta }_{0}$) species mean abundance, ($\beta$) effect of covariate ${X}_{i}$ on species abundance; (${\varphi }_{0}^{DSopen};{\varphi }_{0}^{DS}$) depicting mean species availability estimated by, respectively the structured and semi-structured dataset; ($\gamma$) effect of covariates ${U}_{i,j}$ and ${V}_{i}$ over species availability; (${\sigma }_{0}$) mean species detectability and ($\alpha$) effect of covariate ${Z}_{i,j}$ over species detectability, see Box 1 and Eq. (1). We also included residual errors on species abundance and species detectability, respectively (${\varepsilon }_{i}^{abund}$; ${\varepsilon }_{i}^{det}$) generated from a normal distribution of mean 0 and standard deviation (${\sigma }_{{\varepsilon }^{abund}}$; ${\sigma }_{{\varepsilon }^{det}}$).

1 $$\left\{\begin{array}{c}\begin{array}{c}\text{log}\left({\lambda }_{i}\right)={\beta }_{0}+\beta *{X}_{i}+{\varepsilon }_{i}^{abund} \\ logit\left({\varphi }_{i,j}^{DSopen}\right)= {\varphi }_{0}^{DSopen}+\gamma *{U}_{i,j} \\ logit\left({\varphi }_{i}^{DS}\right)={\varphi }_{0}^{DS}+\gamma *{V}_{i}\end{array}\\ \text{log}\left({\sigma }_{i,j}\right)={\sigma }_{0}+\alpha *{Z}_{i,j}+ {\varepsilon }_{i,j}^{det}\end{array}\right.$$

We used an altered version of the function simHDSopen from *AHMbook*^45^ to simulate the datasets. All models were fitted using JAGS 4.3.1^46^ through the *jagsUI*^47^ R package, while MCMC samples were retrieved using *mcmcoutput*^48^. See appendix S1 for MCMC parameters and priors used for simulation and case study.

### Simulation study 2: estimates accuracy across different sampling scenarios

**Fig. 2:**
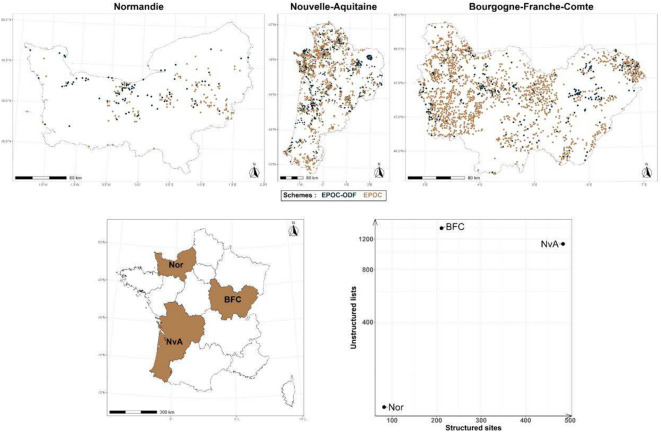
Spatial distribution and repartition of structured sites (EPOC-ODF) and semi-structured lists (EPOC) over selected French regions (*Nor* Normandie, *NvA* Nouvelle-Aquitaine, *BFC* Bourgogne-Franche-Comté). Maps were created using R software version 4.3.1.

In simulation study 2, we aimed to assess improvement in accuracy of estimated parameters through data integration across multiple sampling scenarios. We used the same 1000 cases generated in simulation study 1, but varied the number of structured sites (ranging from 50 to 300) and semi-structured lists. The latter was determined by the multiplication of the number of structured sites, using a ratio ranging from 1 to 6. We defined ranges of the number of structured sites and ratio of added semi-structured lists based on the proportion of sampling schemes in our case study, see Fig. 2 and appendix S2. Inference improvement was associated with a reduction of uncertainty (*i.e*. reduction of the posterior distribution spread of estimated parameters using the 95% credible intervals CRI).

We calibrated a linear model of the log-transformed CRI width to assess if its reduction was affected by factors such as the model formulation used (either HDS or IDS) or estimated parameters. As we expect that model formulation could benefit from the number of input data, we included an interaction between model formulation and the simulated sampling design, *i.e.* the number of simulated structured sites and the ratio of added semi-structured sites. As the response variable of our intended model is derived from simulation results, we conducted a bootstrap to assess variation of CRI reduction through resamples over simulated cases and their associated parameters. Confidence intervals were estimated using 100 linear models, each based on resamples of 250 from converging IDS and HDS models.

### Case study

We relied on EPOC-ODF (Structured Estimation of Common Bird Population Size) and EPOC (Estimation of Common Bird Population Size) citizen science schemes data collected over 2021–2023 breeding seasons. These two schemes consist of 5-min point count completed checklists, during which observers point locations of detected individuals using the mobile app NaturaList^49^. Observation distances between observers and detected individuals are measured through GIS (Geographic Information System) using observers location determined by GPS. We used data from 31 bird species collected during their breeding season over 2021–2023 across three French regions (Bourgogne-Franche-Comté, Nouvelle-Aquitaine and Normandie). These regional datasets differ in terms of data quantity providing diverse distributions of structured and semi-structured data collections (Fig. 2.).

The EPOC scheme does not constrain observers to pre-selected sites, nor require repeated visits whereas, for EPOC-ODF, survey locations are randomly selected from a systematic grid and have to be visited three times during the breeding season, each session consisting of three successive 5-min point counts. For the semi-structured dataset (EPOC), we applied a spatial filter to select EPOC lists collected at least two kilometres away from sites with temporal replicates (EPOC-ODF) and other EPOC lists, see appendix S2. For each species, we calibrated a HDS model, using only data collected by the EPOC-ODF schemes and an IDS model using data collected by both schemes.

Bird species selection was based upon targeted species from the two schemes^33^ and had a sufficient number of observations, at least detected once at 20 distinct EPOC-ODF sites, in each region. We applied a temporal filter that considered both observed bird activities during the breeding season and expert knowledge to define the breeding phenology of each targeted species and exclude potential early or late migrants. For each species, we applied a right-side truncation of 5% over the observation distance to remove extreme distance values for model robustness^50^.

**Fig. 3:**
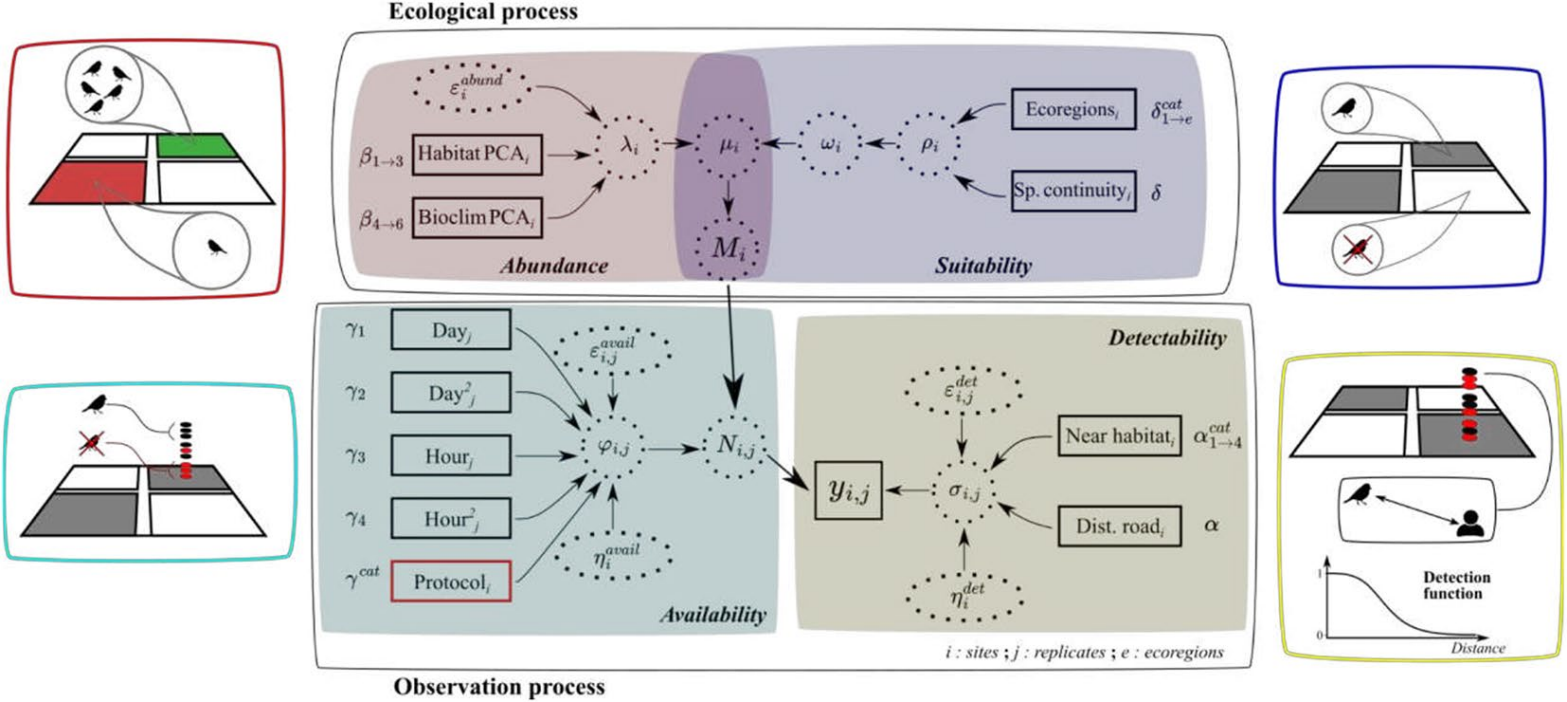
Directed acyclic graph (DAG) representation of the hierarchical model. Observed and latent variables are represented using solid squares and dotted circles respectively. Arrows depicted links between parameters and covariates. Estimated coefficients are depicted on the side of covariates. We include the protocol origin as a categorical covariate, represented by a red box, solely for the IDS model. Each sub-process is represented by distinctive colours and pictograms, from left-to-right and up-to-down we depicted processes (i) describing variation of species abundance across space in relation to habitat covariates; (ii) representing sites’ probability of being considered unsuitable for modelled specie; (iii) assessing species probability of being exposed to sampling occasions and (iv) depicting species probability of being detected given its observation distance.

We modelled the population size of a site ${M}_{i}$ using a Zero-inflated Poisson with parameter ${\mu }_{i}$ (Fig. 3):

$${M}_{i}\sim Poisson\left({\mu }_{i}\right), with \ {\mu }_{i}={\lambda }_{i}*(1-{\omega }_{i})$$

The expected species abundance parameter (${\lambda }_{i}$) was modelled using reduced habitat^51^ and bioclimatic^52^ covariates obtained through PCA^33^.

$$log\left({\lambda }_{i}\right) ={\beta }_{0}+{\sum }_{a=1}^{3}{\beta }_{a}*Habitat PCA{s}_{i}+{\sum }_{a=4}^{6}{\beta }_{a}*Bioclimatic PCA{s}_{i}+{\varepsilon }_{i}^{abund}$$

The zero-inflation parameter (${\omega }_{i}$) corresponds to site suitability depicted by a Bernoulli process with the probability (${\rho }_{i}$) of a site being considered unsuitable. We modelled ${\rho }_{i}$ in regards to site ecoregions, as a categorical variable^53^ and its spatial continuity^54^. We also included a site random effect for abundance (${\varepsilon }_{i}^{abund}$).

$${\omega }_{i}\sim Bernoulli({\rho }_{i})$$

$$logit\left({\rho }_{i}\right)={\rho }_{0}+{\sum }_{a=1}^{e}{\delta }_{a}^{cat}*Ecoregio{n}_{i}+\delta *Spatial\ continuit{y}_{i}$$

From the sampling scheme and temporal intervals between EPOC-ODF sessions, we considered that species availability primarily reflected spatial temporary emigration, due to migratory arrivals and departures during breeding seasons, potentially affecting the number of individuals potentially present on sites during surveys. Consequently, we modelled the probability of an individual being available for detection (${\varphi }_{i,j}$) using covariates such as hour from sunrise and julian date with quadratic effect to represent birds’ phenology across the breeding season. In the IDS model, we included a categorical covariate (${\gamma }^{cat}$) to account for variations in species availability due to the difference of temporal sampling over breeding seasons of the two schemes.

$$logit\left({\varphi }_{i,j}\right)={\varphi }_{0}+{\gamma }_{1}*Da{y}_{j}+{\gamma }_{2}*Da{y}_{j}^{2}+{\gamma }_{3}*Hr.su{n}_{j}+{\gamma }_{4}*Hr.su{n}_{j}^{2}+{\varepsilon }_{i,j}^{avail}+ {\eta }_{i}^{avail}$$

For species detectability, we used a half-normal detection function with parameter (${\sigma }_{i,j}$), where we modelled observers detection probabilities in regards to observed distances using categorical variables describing the habitat over four categories (Agricultural, Forest, Open and Urban;^33^) as well as the distance between their GPS locations and the nearest road^55^. For the IDS model, we considered two distinct intercepts allowing calibration of two separate detection functions, one for each dataset.

$$log \left({\sigma }_{i,j}\right) ={\sigma }_{0}+\alpha *Dist.Roa{d}_{i}+{\sum }_{a=2}^{4}{\alpha }_{a}^{cat}*Near\ habita{t}_{i}+{\varepsilon }_{i,j}^{det}+{\eta }_{i}^{det}$$

For species availability and detectability; we accounted for the study design of the structured dataset by implementing random effects over each session (${\varepsilon }_{i,j}^{avail}$ and ${\varepsilon }_{i,j}^{det}$) while also adding observers random effect over surveyed sites or lists (${\eta }_{i}^{avail}$ and ${\eta }_{i}^{det}$), as one observer can partake in both CS schemes, see appendix S1 for used priors.

We fitted two linear mixed-effects models for assessing CRI reduction and shift in means of estimated parameters between the IDS and HDS. For both linear models, we considered a fixed effect of estimated parameters and included an interaction between model formulation and studied regions. We also added nested random effects over species and studied regions to account for specific species response for each region. Models were fitted using *lme4*^56^. We used *emmeans*^57^ to estimate marginal means from the linear model and pairwise post hoc multiple comparisons. For the case study analysis, we removed (${\gamma }^{cat}$) and (${\delta }^{cat}$) parameters from comparison as the ${\gamma }^{cat}$ is not estimated in the HDS formulation and ${\delta }^{cat}$ parameters varied across studied regions.

## Results

### Simulation study

#### Simulation study 1

**Fig. 4:**
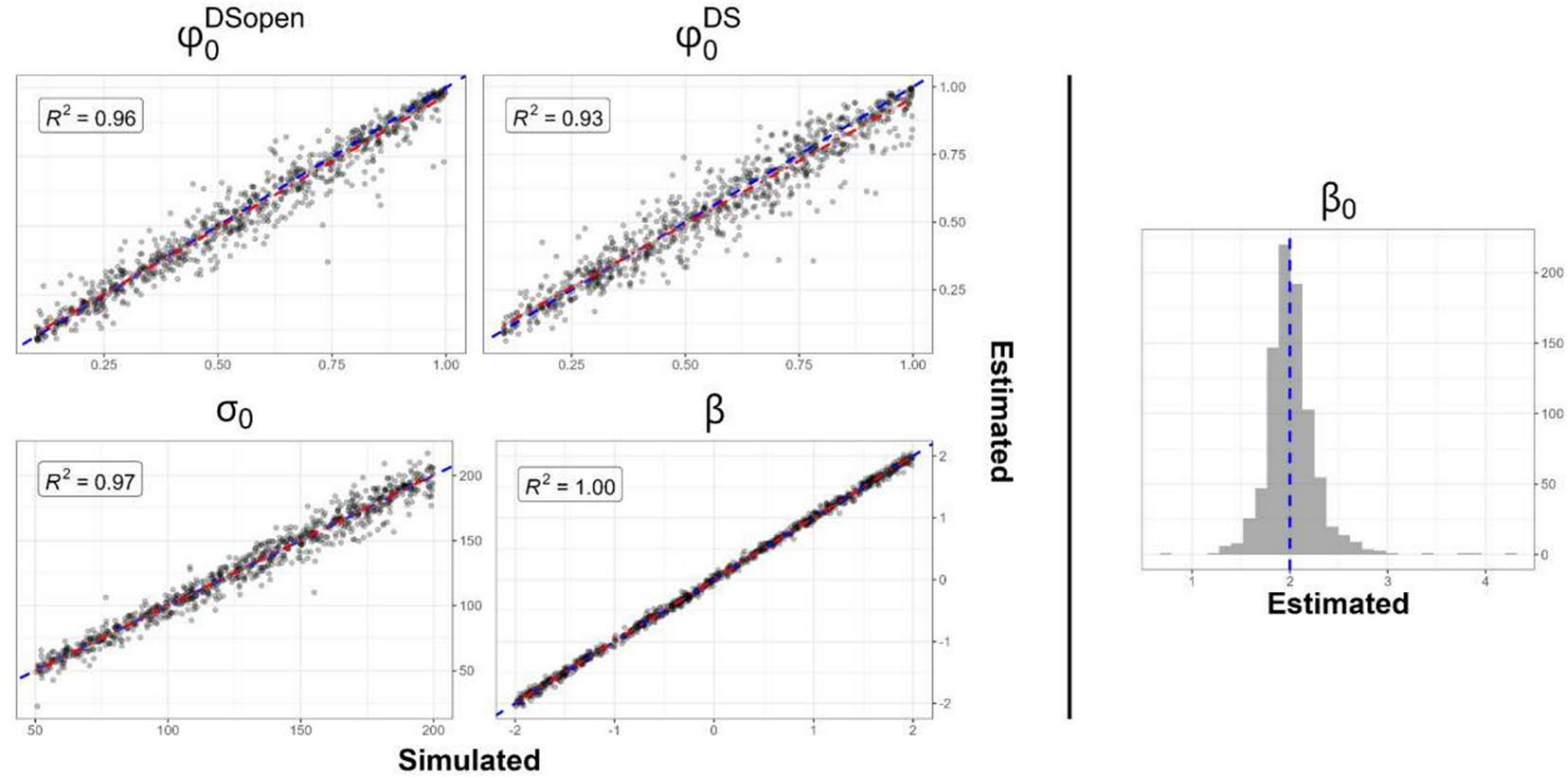
Identifiability plot of converged model for the simulation study 1 for ${\varphi }_{0}^{DSopen}$; ${\varphi }_{0}^{DS}$; ${\sigma }_{ 0}$; $\beta$; ${\beta }_{0}$ and their associated linear regression (dotted red line) R^2^ values. Accurate parameter identification is represented by a dotted blue line. The ${\beta }_{0}$ parameter is depicted as a histogram of estimated values, as we didn’t vary it across simulations. See appendix S4 for identifiability plots of the other generated parameters.

For simulation study 1, 861 out of 1000 simulated datasets resulted in converging models. Overall, the IDS model demonstrated its ability to accurately estimate the parameters for both the ecological and observation processes. While ${\varphi }_{0}^{DSopen}$, ${\varphi }_{0}^{DS}$, ${\sigma }_{{\varepsilon }^{abund}}$ and ${\sigma }_{{\varepsilon }^{det}}$ parameters appeared to have lower precision, all parameters had a coefficient of correlation (R^2^) above 0.85 between their simulated and estimated values (Fig. 4; S4.1). We also see that estimation of ${\beta }_{0}$ were centered over the generated value across all simulation. See appendix S3 for an analysis of model convergence of simulation studies.

#### Simulation study 2

**Fig. 5:**
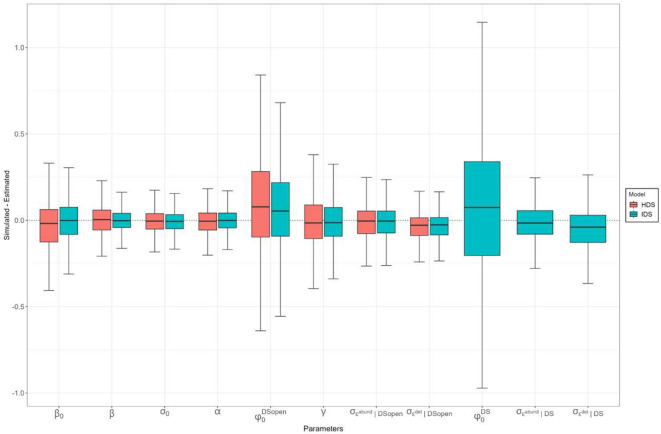
Boxplot of differences (simulated—estimated) across mean estimated parameters for the HDS and IDS models. Parameters are represented in their respective scale (log or logit). Accurate estimation, *i.e*. no bias, is depicted by the dotted line. ${\varphi }_{0}^{DS}$ and standard deviations of residual errors of the semi-structured schemes (DS) were only estimated in the IDS model.

**Fig. 6:**
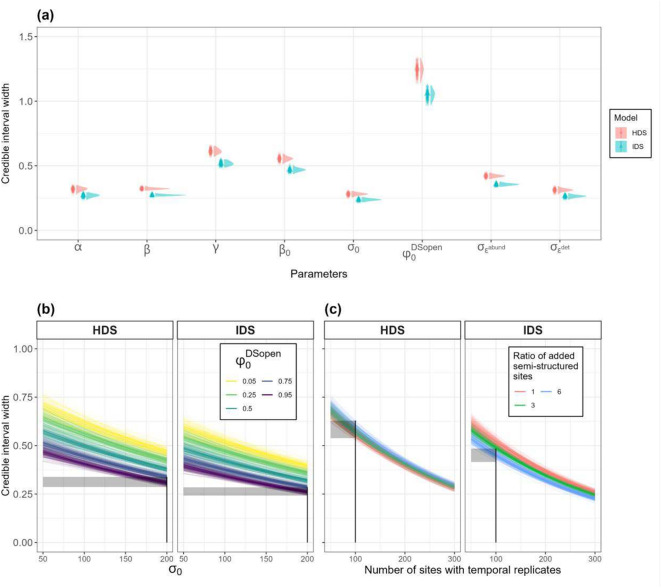
Marginal plot the simulation study 2 representing credible intervals (CRI) width obtained from the HDS and IDS models over 844 simulations. (a) Average CRI and their associated bootstrapped confidence intervals (CIs), depicted by vertical density plots, over simulated parameters. (b) CRI average responses and CIs over simulated species availability (${\varphi }_{0}^{DSopen}$) and detectability (${\sigma }_{0}$) continuums on natural scales. Species availability averaged across multiple classes (0.05, 0.25, 0.5, 0.75 and 0.95 detection probability) are depicted by colour-graded lines. (c) CRI average responses and CIs over multiple data collection cases. Ratio of semi-structured data (without temporal replicates) averaged over three classes (1,3 and 6) are depicted by colour-graded lines. Each dot (**a**) and line (**b**,**c**) correspond to a model marginal response from a bootstrapped resample consisting of 250 randomly selected converged models, allowing visualisation of the response signal CIs. For visual comparison between the HDS and the IDS estimates accuracy, we plotted CRI responses, grey ribbons, depicting the case of simulated species with high mean detectability (${\sigma }_{0}$ = 200 m) and high probability of being available (${\varphi }_{0}^{DSopen}$ = 0.95) surveyed over 100 sites with temporal replicates and six times the number of added semi-structured sites (c), depicted with vertical lines. Lower and upper bounds of the rectangles correspond to minimal and maximal estimated CRI width values.

For simulation study 2, out of 1000 simulated datasets, we had 892 converging models using the HDS formulation and 930 converging models using the IDS formulation. There were no signs of major bias between simulated and the mean of parameter estimates considered (Fig. 5). Bootstrap resamples were based on 844 converging models for both the HDS and IDS formulation. We obtained a considerable reduction of CRI width across all estimated parameters for the IDS model (Fig. 6a). Overall, the IDS and the HDS models produced narrower CRI for available and more easily detectable species, however, the IDS model produced narrower CRI, for equivalent species availability-detectability profiles simulated than the HDS (Fig. 6b). The number of structured sites, i.e. including temporal replicates, was correlated with a reduction of CRI width for both models (Fig. 6c), although the IDS model CRI reduction was also correlated with an increasing proportion of semi-structured sites added to the calibration dataset (Fig. 6c). While the increasing proportion of semi-structured sites added to the calibration dataset had no substantial effect on the HDS model, we found an important correlation to a CRI reduction for the IDS model (Fig. 6c). See Appendix S6 for a comparison of CRI reduction in simulation study 2, where three temporal replicates were considered instead of nine.

### Case study

**Fig. 7:**
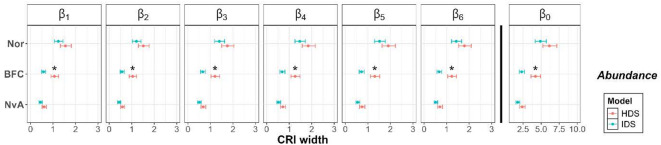
Marginal effect plots of CRI widths of estimated parameters and their associated CIs, in regards to the model (IDS in blue and HDS in red) and French regions (Nor: Normandie; NvA: Nouvelle-Aquitaine and BFC: Bourgogne-Franche-Comté). Coefficients of habitat effects on species abundance (${\beta }_{1\to 6}$) are depicted to the left of the vertical black line, while mean abundance (${\beta }_{0}$) is represented on the right. Significant gaps of averaged mean estimated parameters between models are highlighted with an asterisk. See appendix S5 for additional marginal effect plots for parameters associated with suitability, availability and detectability.

Marginal effect plots from the linear model (Fig. 7) showed that Credible Intervals (CRI) were slightly wider for Normandie (Nor), the region with fewer structured sites and semi-structured sites than Bourgogne-Franche-Comté (BFC), the region with a few numbers of structured sites and a large number of semi-structured sites, and Nouvelle-Aquitaine (NvA), the region with a larger number of semi-structured sites (Fig. 2). While there were no considerable differences (indicated by an overlap of marginal response confidence intervals) between the HDS and IDS CRI for NvA and Nor, CRI from the IDS model were considerably narrower than the HDS ones for all estimated parameters in BFC (Fig. 7 and appendix S5). Pairwise comparison of marginal means showed no signs of significant differences (p-values > 0.05) between the HDS and IDS mean estimated parameters across all monitored parameters and studied regions. Squared-GVIFs (Generalised Variance-Inflation Factor; Ref.^58^), measured using *car* R package^59^, were less than 4, showing no signs of multicollinearity for the terms used in each model.

## Discussion

The present work brings new evidence that Integrated Distance Sampling (IDS) models can accurately identify parameters of a complex ecological process and expand their application accounting for species availability determined through repeated visits. Moreover, it also shows that data integration improves ecological inference, through the reduction of credible intervals (CRI) width, for all parameters of the studied ecological process, across multiple sampling design scenarios and species availability-detectability continuums. Results from the case study further strengthen the simulation study, by showing that this reduction of CRI span without significant variations of estimated mean parameters depends on the ratio of structured and semi-structured data used for each case study.

In recent years, there has been an increase in the interest for integrated models^26,27^ due to their efficiency in reducing potential biases inherent to a single dataset^60^ and allowing reliance on automated and non-invasive data collection methods^61,62^. Data integration through joint likelihood^63^ still has potential drawbacks when temporal and/or spatial mismatches, corresponding to discontinuity between dataset timeframes and spatial heterogeneity, are unaccounted for. Such mismatches could lead to biased inferences where the sampled timeframes and/or regions do not correctly represent the ecological process of interest^63,64^.

In our simulation studies, we did not include spatial bias in data collection, which could potentially misrepresent citizen science spatial sampling bias^65^. We accommodated this mismatch in the case study through a spatial filter over the semi-structured dataset based upon the 2 × 2 km systematic grid resolution of the structured scheme. This resulted in an important decrease in available data from the semi-structured dataset (see appendix S2) that could be resolved through random effects in the model^27^. In our case, we could consider distinct spatial subsets of the semi-structured dataset and implement them into a random effect structure encompassing the modelled sub-processes of the integrated model.

While hierarchical models offer a viable option to disentangle variations due to the observation process from the variations originating from the ecological process of interest^9^, the trade-off between data specificities and data quantity can limit their applications. Data integration corresponds to a valuable option to increase the number of available data to help calibrate such models. Data integration also needs to account for sampling schemes specificities and their potential effect on estimated parameters. For instance, the variation of species availability in regards to list duration between standardised schemes and non-standardised schemes with varying durations^38^. Options to calibrate integrated hierarchical models exist in a frequentist framework^38^ allowing fast computation. However, given the types of available data and ecological processes of interest, data integration is prone to rely on Bayesian frameworks. Bayesian computation is based on Markov chain Monte Carlo (MCMC) techniques which are computationally intensive^66^. Novel approaches exist such as Integrated Nested Laplace Approximation (INLA) or Bayesian emulation^67,68^ allowing efficient computation and facilitating implementation of spatial components^69^.

Integrated models could represent an important tool for macro-ecology related studies, spanning across large spatial scales^27^ or requiring multiple institutions to coordinate data collection^70,71^. It could be used, for instance, in the study of bird populations across Europe from the pan-european common bird monitoring (PECBMS), which gathers data from point count, line transect, or territory mapping schemes across 28 countries and varying numbers of fieldworkers^72^, while taking account of country discrepancies in sampling design, sampling effort or varying starting period that could alter estimation of long-term trend^73^. The joint analysis of multiple data sources, notably through the use of data collected upon schemes lacking design-based methodology^74^, could represent a substantial increase in the quantity of data available for the study of cryptic species^75,76^ and improve assessment of migratory patterns over large spatial scales^77^. It represents an influx of data for the estimation of ecological processes of interest^27^, potentially reducing the sampling effort of robust designs. For instance, the number of temporal replicates considered in simulation studies and case study exceeds that of most commonly used schemes. To assess data integration utility beyond our specific case, we conducted an additional simulation study considering a structured scheme composed of three temporal replicates instead of nine (see appendix S6). Comparison of the HDS and IDS formulations over both temporal replicate quantities revealed that data integration had a greater effect in parameter accuracy when applied to the less demanding structured survey. However, it remained less accurate than estimates derived from using only data collected from the structured scheme with nine temporal replicates (Figure S6.1–3). Before their implementation, we highly recommend assessing whether ‘lessen’ structured sampling designs developed in a data integration context are still capable of estimating the targeted ecological parameters or only partially, using power analysis^78^ and assessment of integrated models identifiability via simulations^79^.

Our results highlight the benefits of relying on statistical frameworks such as Integrated Models capable of improving estimates accuracy through expansion of usable data collected from structured and semi-structured surveys. While our simulation results showed a constant reduction of estimates uncertainty, results from field surveys in three distinct French regions, depicting distinct ratios in quantity of structured and semi-structured data, showed that this improvement is case-dependant and significantly reduced estimates uncertainty with a low quantity of structured data and high quantity of semi-structured data. While we advocate for thorough planning before sampling, this suggests that Integrated Models could represent a conceivable alternative in case of insufficient collection from structured surveys and could also greatly benefit from data collected by citizen science schemes.

## Supplementary Information

Supplementary Information.

## Data Availability

Scripts, BUGS model files and data for simulation and case studies replications are available online: 10.5281/zenodo.11452853
