# Supplementary material for: Improving national-scale breeding bird surveys with integrated distance sampling
Source: Sci Rep. 2025 May 26;15:18312. doi: 10.1038/s41598-025-96787-w (PMC12106623; doi:10.1038/s41598-025-96787-w)
Supplement: Supplementary file 1 — Supplementary Information. [file 41598_2025_96787_MOESM1_ESM.pdf]

Improving national-scale Breeding Bird Surveys with Integrated Distance Sampling

Jean Nabias[1,2,*], Romain Lorrillière[2,3], Jérémy Dupuy[1], Laurent Couzi[1], Luc Barbaro[2,4]

1 : *LPO-BirdLife France, Fonderies Royales, Rochefort Cedex, France*

2 : *CESCO, Muséum National d'Histoire Naturelle, CNRS, Sorbonne-University, Paris, France*

3 : Centre de Recherches sur la Biologie des Populations d'Oiseaux (CRBPO), MNHN-CNRS-OFB, Paris, France

4 : *Dynafor, INRA-INPT, University of Toulouse, Auzeville, France*

ORCID

Jean Nabias : 0009-0000-8864-0165

Romain Lorrillière : 0000-0003-1870-0278

Luc Barbaro : 0000-0001-7454-5765

Keywords: Bird monitoring ; Citizen science ; Distance sampling ; Data integration ; Hierarchical modelling ; Observation process

IMPROVING NATIONAL-SCALE BREEDING BIRD SURVEYS WITH INTEGRATED DISTANCE SAMPLING **1**

S1 - MCMC parameters and priors **3**
Simulation studies 1 and 2 3
Case study 4

S2 - Semi-structured dataset spatial filter **6**

S3 – Model convergence analysis for the simulation studies **7**

S4 - Simulations identifiability: Complementary figures **10**
Simulation 1: Coefficients identifiability 10
Simulation 2: Identifiability 11

S5 – Case study: Additional marginal effect plots **13**

S6 – Effect of the number of temporal replicates **14**

S1 - MCMC parameters and priors

Simulation studies 1 and 2

MCMC parameters

Iteration: 60.000

Burn-in: 12.000

Thin: 15

Chains: 4

Range for species parameter generation

$\overline{\varphi_0^{DSopen}} \sim U(0,1)$ and $\varphi_0^{DSopen} = logit(\overline{\varphi_0^{DSopen}})$

$\overline{\varphi_0^{DS}} \sim U(0,1)$ and $\varphi_0^{DS} = logit(\overline{\varphi_0^{DS}})$

$\overline{\sigma_0} \sim U(50,200)$ and $\sigma_0 = logit(\overline{\sigma_0})$

$\gamma \sim U(-2,2)$

$\alpha \sim U(-2,2)$

$\beta \sim U(-2,2)$

$\sigma_{\varepsilon abund} \sim U(0.1,1)$

$\sigma_{\varepsilon det} \sim U(0.1,1)$

Specific for the simulation study 2:

Number of structured sites (with temporal replicates) $\sim U(50,3000)$, rounded to the first decimal.

Ratio of semi-structured lists and structured sites $\sim U(1,6)$

MCMC Priors

$\bar{\beta}_0 \sim U(0,10)$ and $\beta_0 = \log(\bar{\beta}_0)$

$\overline{\varphi_0^{DSopen}} \sim U(0,1)$ and $\varphi_0^{DSopen} = logit(\overline{\varphi_0^{DSopen}})$

$\overline{\varphi_0^{DS}} \sim U(0,1)$ and $\varphi_0^{DS} = logit(\overline{\varphi_0^{DS}})$

$\overline{\sigma_0} \sim U(0,500)$ and $\sigma_0 = logit(\overline{\sigma_0})$

$\alpha \sim N(0,10)$

$\gamma \sim N(0,10)$

$\beta \sim N(0,10)$

$\sigma_{\varepsilon^{abund}} \sim U(0.01,2)$

$\sigma_{\varepsilon^{det}} \sim U(0.01,2)$

Case study

<u>MCMC parameters</u>

Iteration: 350.000

Burn-in: 175.000

Thin: 35

Chains: 4

<u>Priors</u>

<u>*Abundance*</u>

$\beta_0 \sim N(0,10)$

$\tau_{\beta_0} \sim Gamma(1,1)$

$\varepsilon_i^{abund} \sim N(0, \tau_{\beta_0})$, with *i* corresponding to sites

$\beta_{1\rightarrow 6} \sim N(0,10)$

<u>*Suitability*</u>

$\bar{\rho}_0 \sim U(0,1)$ and $\rho_0 = \mathrm{logit}(\bar{\rho}_0)$

$\delta \sim N(0{,}10)$

$\delta^{cat}_{1\rightarrow e} \sim N(0{,}10)$, with *e* corresponding to the number of ecoregions

Availability

$\bar{\varphi}_0 \sim U(0{,}1)$ and $\varphi_0 = \text{logit}(\bar{\varphi}_0)$

$\gamma_{1\rightarrow 4} \sim N(0{,}10)$

$\gamma^{cat} \sim N(0{,}10)$

$\mu_{\eta_o} \sim N(0, \tau^{avail})$, with *o* corresponding to observers

$\eta^{avail}_i \sim N(\mu_{\eta_o}, \tau^{avail}_o)$

$\varepsilon^{avail}_i \sim N(0, \tau^{avail}_1)$

$\varepsilon^{avail}_{i,j} \sim N(\varepsilon^{avail}_i, \tau^{avail}_2)$

τ^{avail}_o, τ^{avail}_1 and $\tau^{avail}_2 \sim Gamma(1{,}1)$

Detectability

$\bar{\sigma}_0 \sim U(0.1{,}500)$ and $\sigma_0 = \log(\bar{\sigma}_0)$

$\alpha \sim N(0{,}10)$

$\alpha^{cat}_{1\rightarrow 4} \sim N(0{,}10)$

$\mu_{\eta_o} \sim N(0, \tau^{det})$

$\eta^{det}_i \sim N(\mu_{\eta_o}, \tau^{det}_o)$

$\varepsilon^{det}_i \sim N(0, \tau^{det}_1)$

$\varepsilon^{det}_{i,j} \sim N(\varepsilon^{det}_i, \tau^{det}_2)$

τ^{det}_o, τ^{det}_1 and $\tau^{det}_2 \sim Gamma(1{,}1)$

S2 - Semi-structured dataset spatial filter

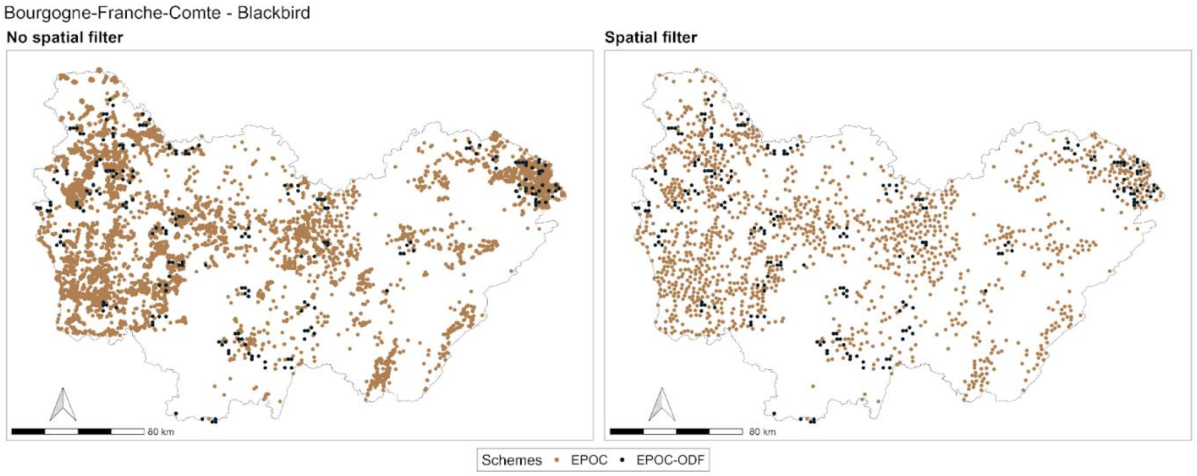

Fig. S2.1: Example of the spatial filter in Bourgogne-Franche-Comte region for the Blackbird (*Turdus merula*).

Observation data collected by birders across the breeding season spanning from March 1st to June 31th. For mountainous regions, a slight shift later in the season is considered (late March to late July).

We first apply a filter over collected lists to target species phenology (available in reproducibility shared data). Spatial filters are specifics of the semi-structured dataset (EPOC). We first select all EPOC lists over 2 kilometres distances of structured dataset (EPOC-ODF) sites. From this first selection, we subsampled EPOC lists spaced by at least 2 kilometres, the resolution of EPOC-ODF sampling design (Appendix S1, Nabias et al., 2024).

Taking the example of the blackbird, prior to spatial filtering, we had 10,670 EPOC lists and 211 EPOC-ODF sites and ended up with 1,385 EPOC lists and 211 EPOC-ODF sites, a reduction of 87% of EPOC usable data.

References:

Nabias, J. et al. Reassessment of French breeding bird population sizes using citizen science and accounting for species detectability. (2024).

S3 – Model convergence analysis for the simulation studies

For simulation studies 1 and 2, we had, respectively, 861 and 844 converging models. As we tested different randomly selected combinations of parameters, we intended to test if model non convergence was more prone to appear at certain values of specified parameters. We considered that converging model as model with $\hat{R}$ value lesser than 1.1. We calibrated a binomial model with the model status (converging or not converging) as the response variable. We included simulated values of all tested parameters as fixed effects.

For the simulation study 2, we calibrated a multinomial model to assess 4 cases for each simulated scenarios: (i) only the HDS model converged; (ii) only the IDS model converged; (iii) both models converged and (iv) none model converged. We used the same fixed effect as the binomial model, but we also included an interaction between the number of given their types, i.e. structured or semi-structured.

Odd ratios of the binomial model (Fig S3.1) showed that simulation study 1 model convergences were positively correlated to φ_0^{DSopen}, φ_0^{DS}, $\sigma_{\varepsilon abund}$ and $\sigma_{\varepsilon det}$. Results for the simulation study 2 showed that 4 out of 10 generated parameters had a significant effect over model convergences. The IDS and HDS models were more prone to converge with higher value of φ_0^{DSopen}, φ_0^{DS}, σ_0 and $\sigma_{\varepsilon det}$ (Fig S3.2). While low values of species availability (φ_0^{DSopen}, φ_0^{DS}) and species detectability (σ_0) can lower the number of collected data between and during surveys thus hindering MCMC convergence capabilities.

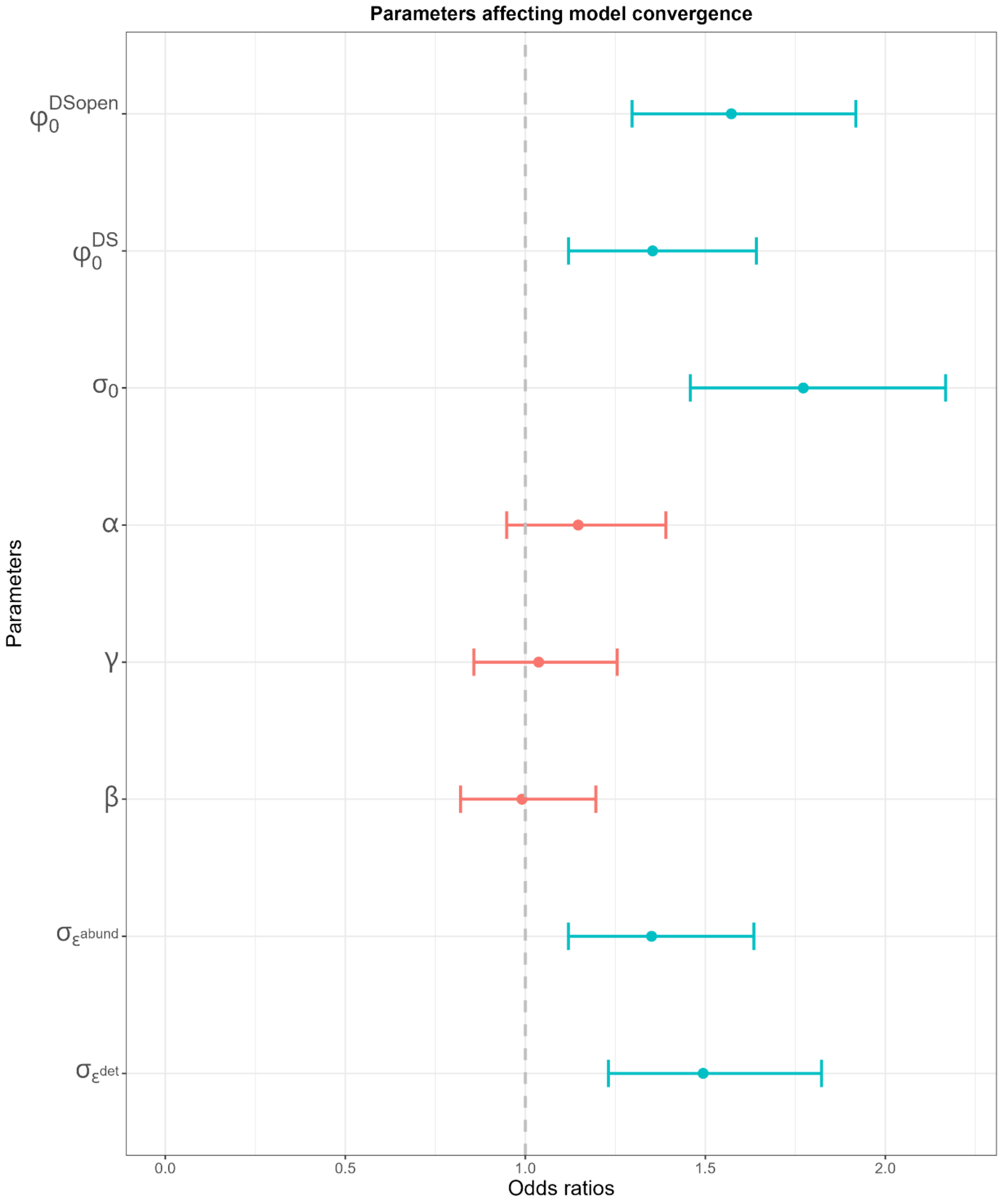

Fig. S3.1: Estimated parameters odd ratios and their confidence intervals. Parameters with significant effect over simulation 1 model convergence are depicted in blue (p-value ≥ 0.05).

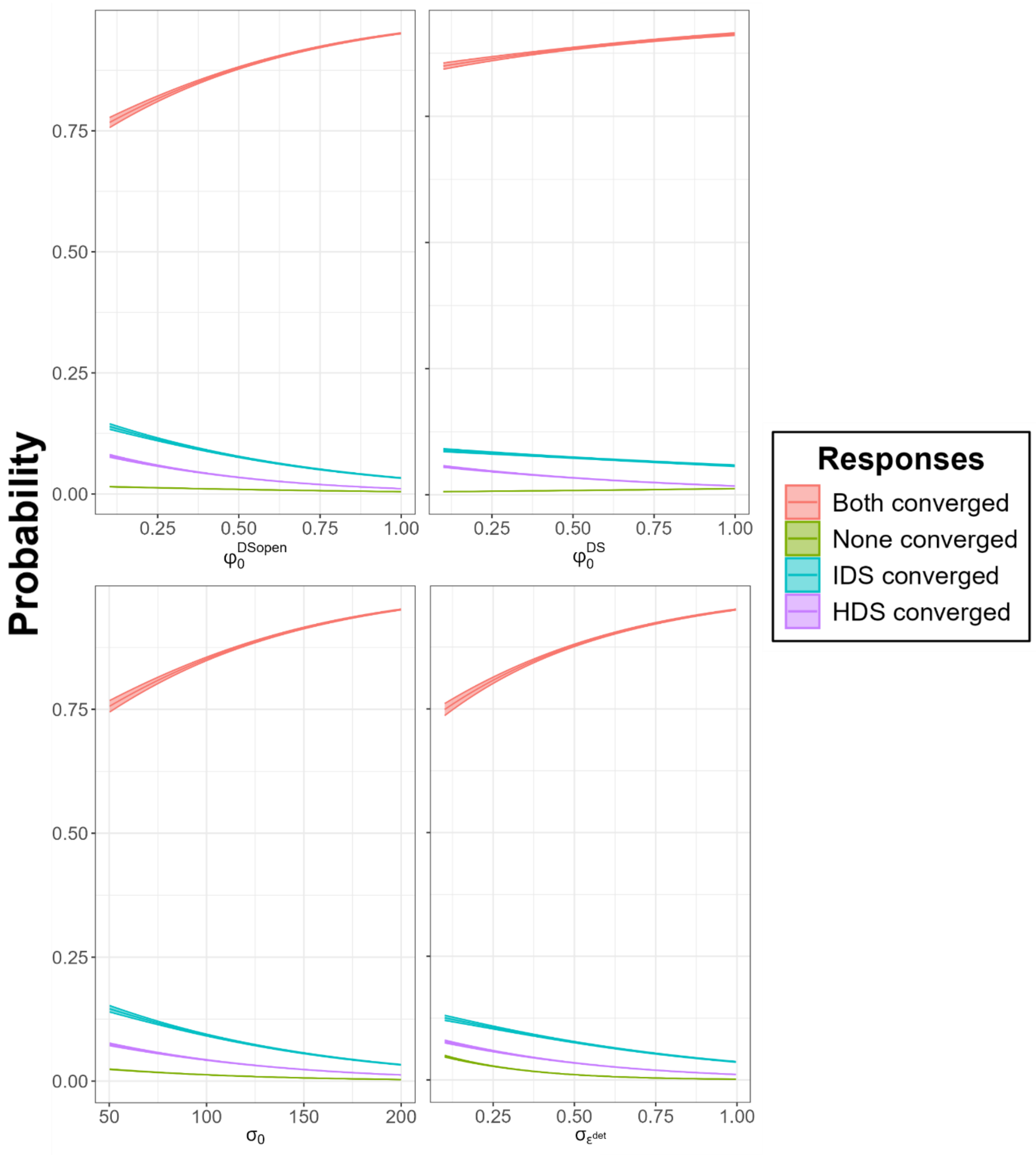

Fig. S3.2: Marginal effect plots of significant parameters affecting model convergence in simulation study 2. Additional marginal plots for all parameters are available in provided data.

S4 - Simulations identifiability: Complementary figures

Simulation 1: Coefficients identifiability

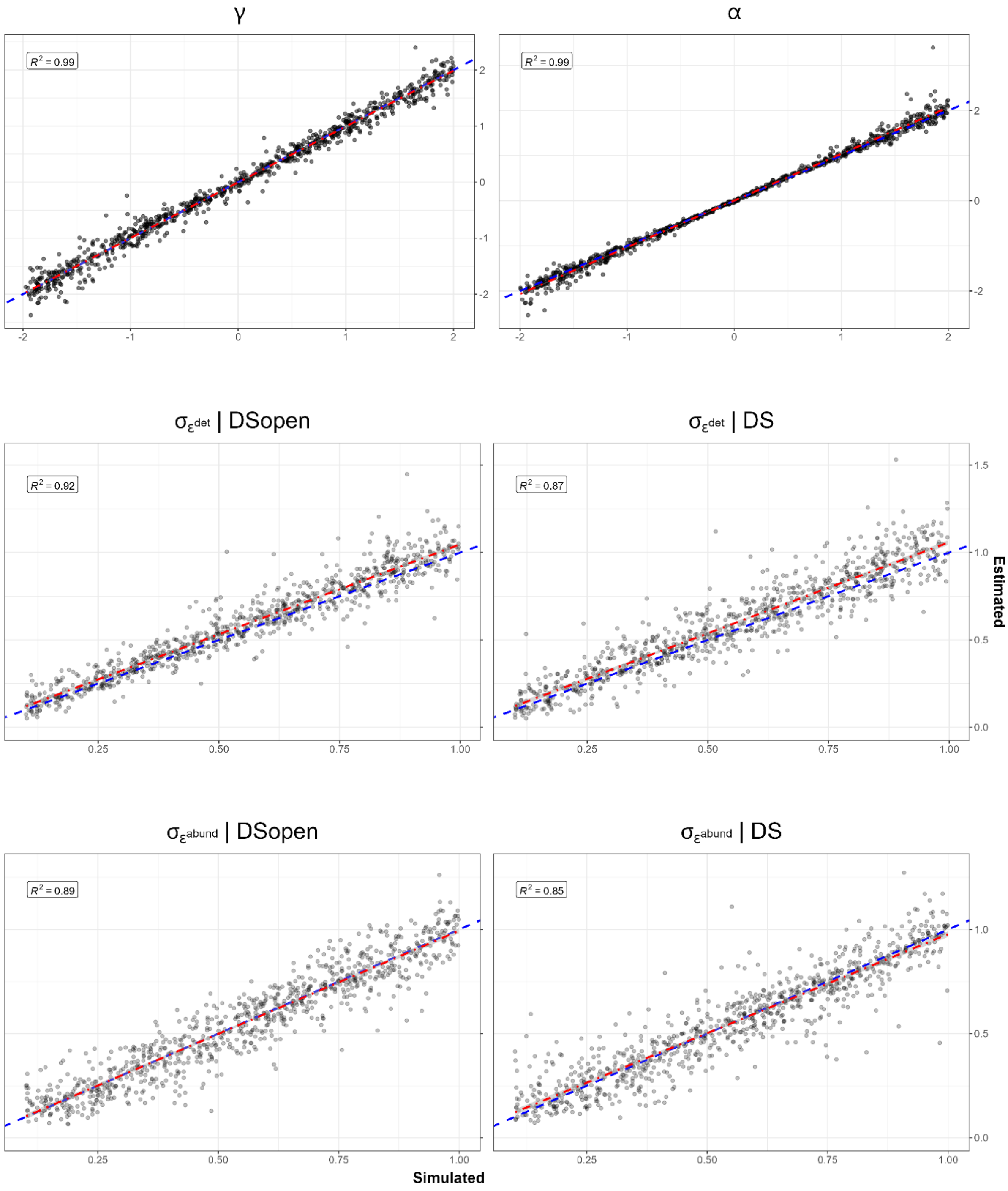

Fig S4.1: Simulation 1 identifiability plot for availability (γ), detectability (α) coefficients, standard deviation of site abundance residuals errors ($\sigma_{\varepsilon abund}$) and site-visit level detectability

($\sigma_{\varepsilon det}$) for structured (DSopen) and semi-structured (DS) datasets. Coefficient of correlation (R^2) values between generated and estimated parameters are depicted in top left corners

Simulation 2: Identifiability

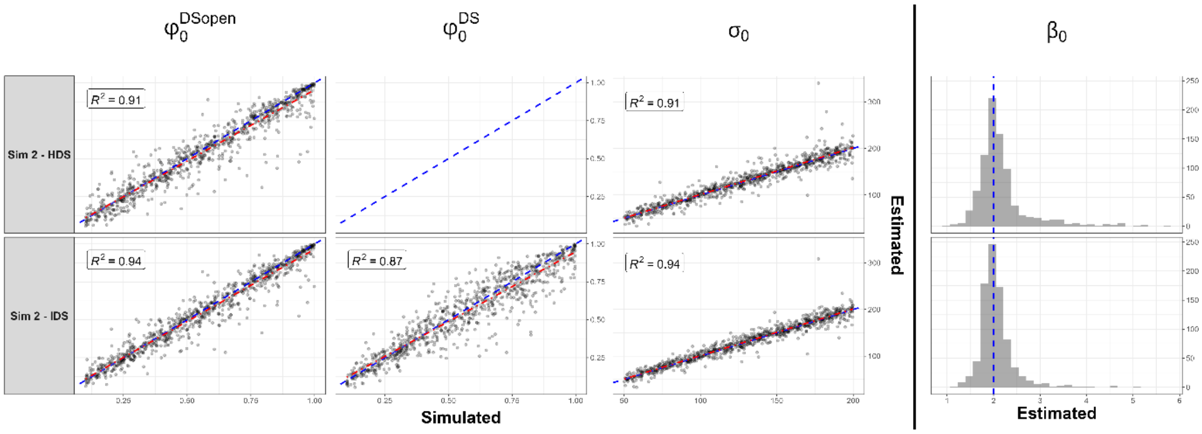

Fig S4.2: Identifiability plot for the simulation study 2 for $\varphi_0^{DSopen}, \varphi_0^{DS}, \sigma_0$ and β_0 with their associated linear regression (dotted red line) R^2 values. Results from the model using only structured data (HDS) are shown in the first row, while results for the model using both structured and semi-structured data (IDS) are shown in the second row. Accurate parameter identification is represented by a dotted blue line. The β_0 parameter is depicted as a histogram of estimated values, as we didn't vary it across simulation.

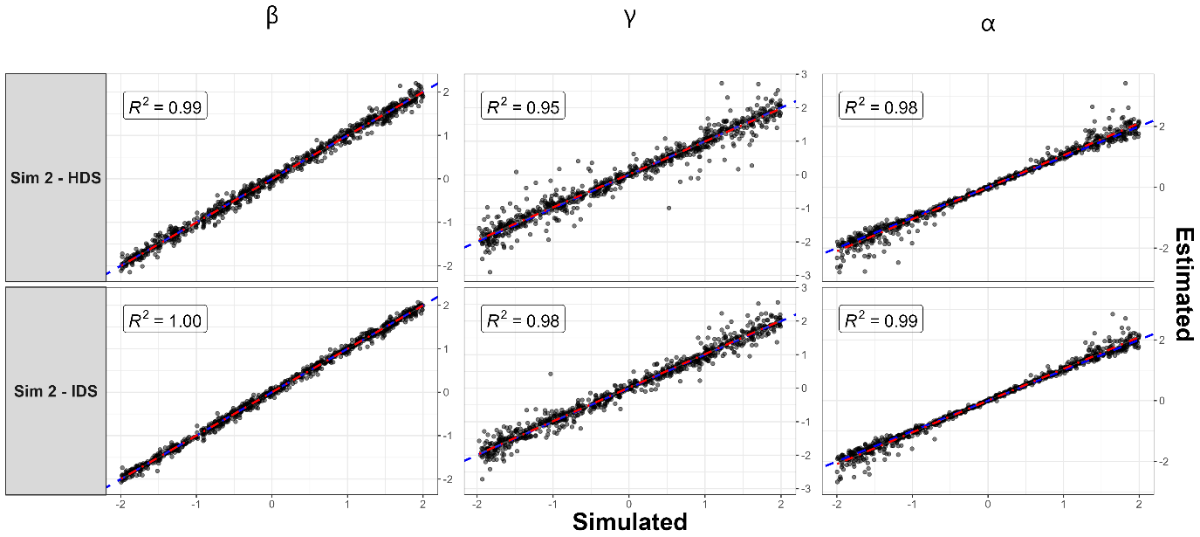

Fig S4.3: Simulation 2 identifiability plot for habitat (β), availability (γ) and detectability (α) coefficients and their associated R^2 values.

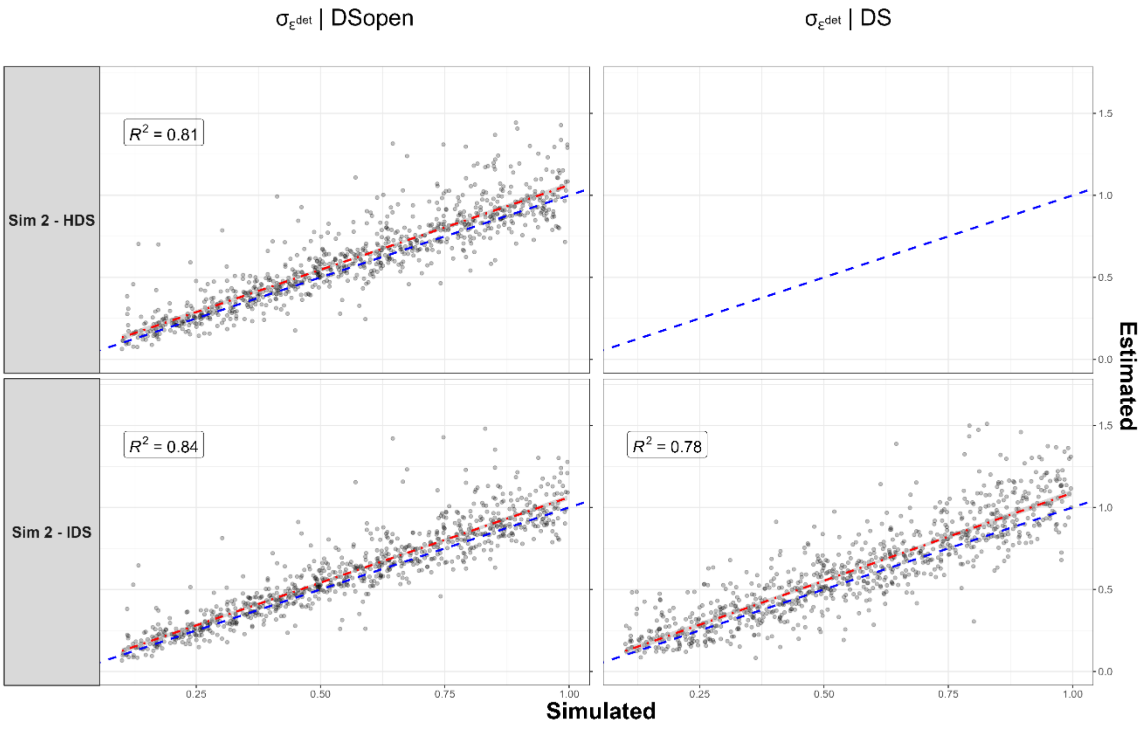

Fig S4.4: Simulation 2 identifiability plot for standard deviation of residuals errors of site-visit level detectability ($\sigma_{\varepsilon det}$) for the structured (DSopen) and semi-structured (DS) schemes

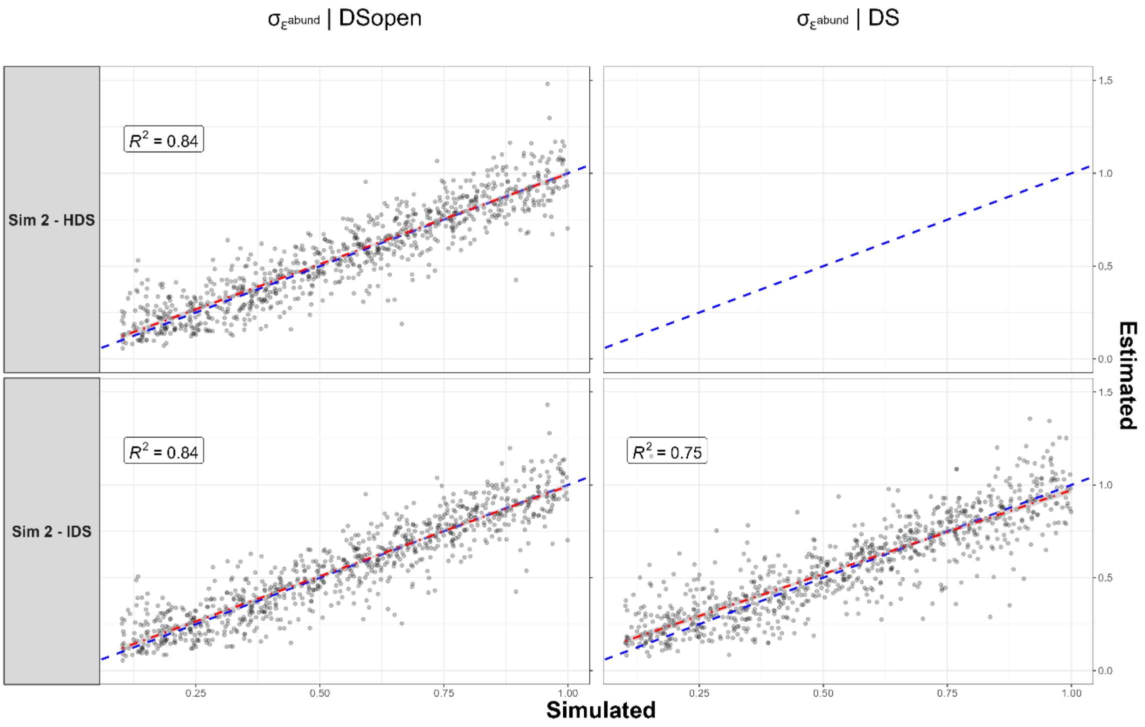

Fig S4.5: Simulation 2 identifiability plot for of site abundance residuals errors ($\sigma_{\varepsilon abund}$) for the structured (DSopen) and semi-structured (DS) schemes

<u>S5 – Case study: Additional marginal effect plots</u>

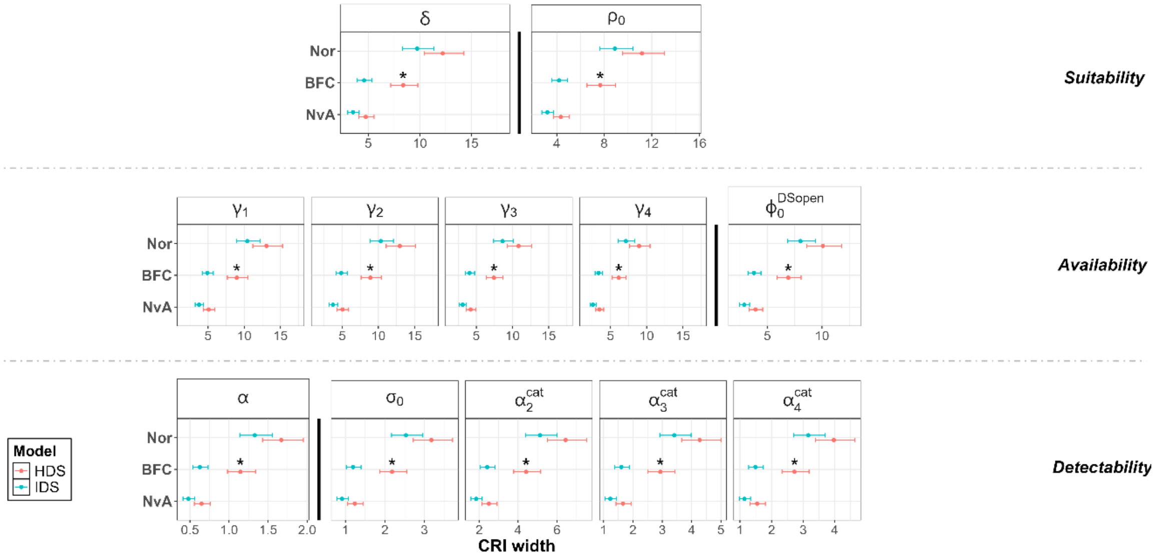

<u>Fig S5.1</u>: Marginal effect plots of CRI widths of estimated parameters and their associated CIs, in regards to the model (IDS in blue and HDS in red) and French regions (**Nor**: Normandie; **NvA**: Nouvelle-Aquitaine and **BFC**: Bourgogne-Franche-Comté). Results for parameters associated with suitability, availability and detectability. In each row, coefficients are depicted to the left of the vertical black line, while intercepts are represented on the right, for species detectability we depicted the intercept (σ_0) as well as its associated categorial effects ($\alpha_{2\to4}^{cat}$). Significant gaps of averaged mean estimated parameters between models are highlighted with an asterisk.

S6 – Effect of the number of temporal replicates

In this supplementary study, we used the same parameters (species and sampling related) as the simulation study 2, but decreased the number of temporal replicates to three. As the number of temporal replicates used in the simulation study and collected by observers in the case study is higher than most structured schemes commonly used.

The main objective of this study is to calibrate a similar linear model as the one used in the simulation study 2 (see Material and methods of the main manuscript) over a dataset comprised of both converging models while implementing an interaction between the fixed effect and categorical covariates splitting IDS model with nine temporal replicates (used in the main manuscript) and IDS model with three temporal replicates. We included this interaction term over all fixed effect as we hypothesized that the number of temporal replicates is linked to the total number of information gathered by the sampling schemes. As such, credible intervals (CRI) estimated by the IDS with three temporal replicates should be wider (i.e. greater uncertainty over estimated parameters) than the IDS with nine temporal replicates.

Out of the 1000 generated datasets consisting of three temporal replicates for the structured scheme, we had 788 converging models with the HDS formulation and 849 converging model with the IDS formulation. Comparing these results to the ones from the main manuscript, we see a clear decrease in the number of converging model (892: HDS; 930: IDS) mainly due to difference in the quantity of temporal replicates. We based our bootstrap on 629 converging model for the HDS and IDS formulations over both cases of temporal replicates. Confidence intervals of figure S6.1 were computed from 100 linear models based on resamples of 200 converging models.

Overall, the number of temporal replicates considered (three or nine) had a significant effect on CRI reduction across all parameters of interests (Fig S6.1), gradients of species availability-detectability (Fig S6.2) and generated sampling scenarios (Fig S6.3). CRI reduction was particularly considerable parameters associated with the availability process (γ and φ_0^{DSopen}; Fig S6.1). Comparing CRI reduction in the highlighted case (grey-ribbons in Fig S6.2 and Fig S6.3) of a highly available and detectable species (φ_0^{DSopen} = 0.95 and σ_0 = 200m) with a sampling scenario of 100 structured sites with six times the amount of semi-structured sites (without temporal replicates), we found that data integration applied to a structured scheme with three temporal replicates produced greater CRI reductions than when used in a scheme with nine temporal replicates. However, it remained less accurate than an HDS model calibrated using a scheme with nine temporal replicates.

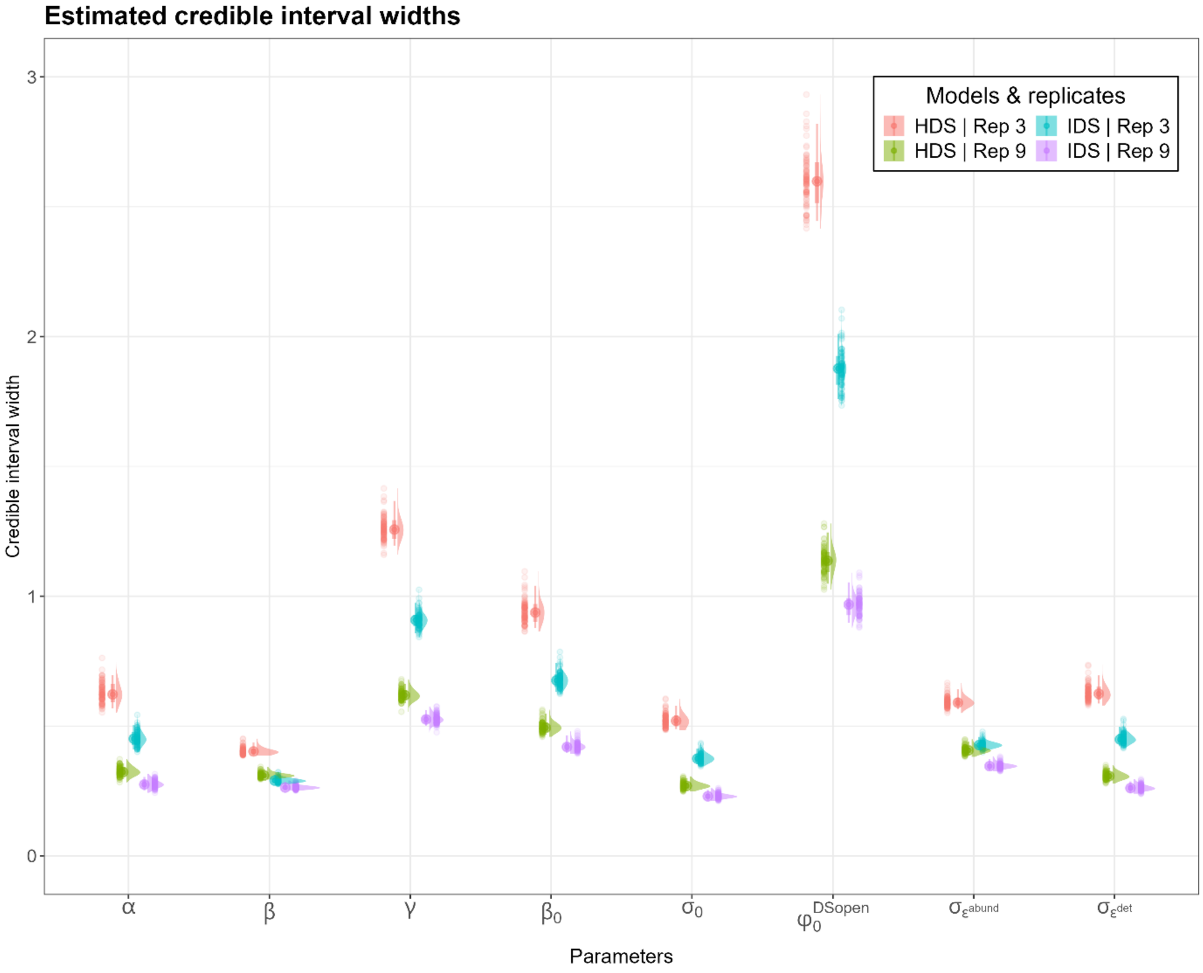

Fig S6.1: Marginal effect plot of CRI reduction across parameters of interests and the number of temporal replicates considered in generated structured datasets. Average CRI and their associated bootstrapped confidence intervals (CIs), depicted by vertical density plots, over simulated parameters.

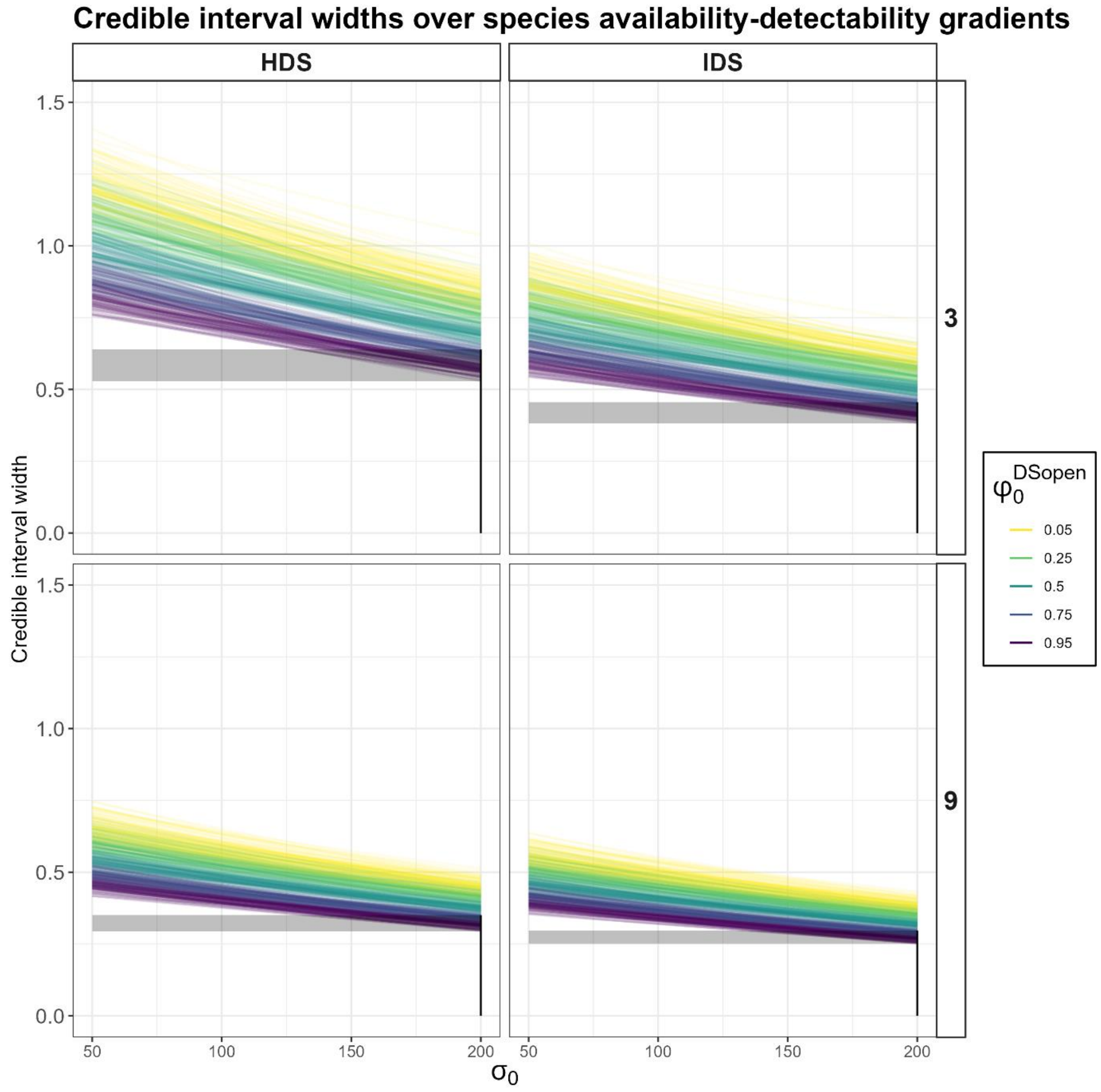

Fig S6.2: CRI average responses and CIs over simulated species availability (φ_0^{DSopen}) and detectability (σ_0) continuums on natural scales. Species availability averaged across multiple classes (0.05,0.25,0.5,0.75 and 0.95 detection probability) are depicted by colour-graded lines. HDS and IDS formulation results are depicted by columns, while the number of temporal replicates considered in simulated structured datasets (three or nine) are depicted by row. For visual comparison between the HDS and the IDS estimates accuracy, we plotted CRI responses, grey ribbons, depicting the case of simulated species with high mean detectability (σ_0 = 200m) and high probability of being available (φ_0^{DSopen} = 0.95) surveyed over 100 sites with temporal replicates and six times the number of added semi-structured sites (c), depicted with vertical lines. Lower and upper bounds of the rectangles correspond to minimal and maximal estimated CRI width values.

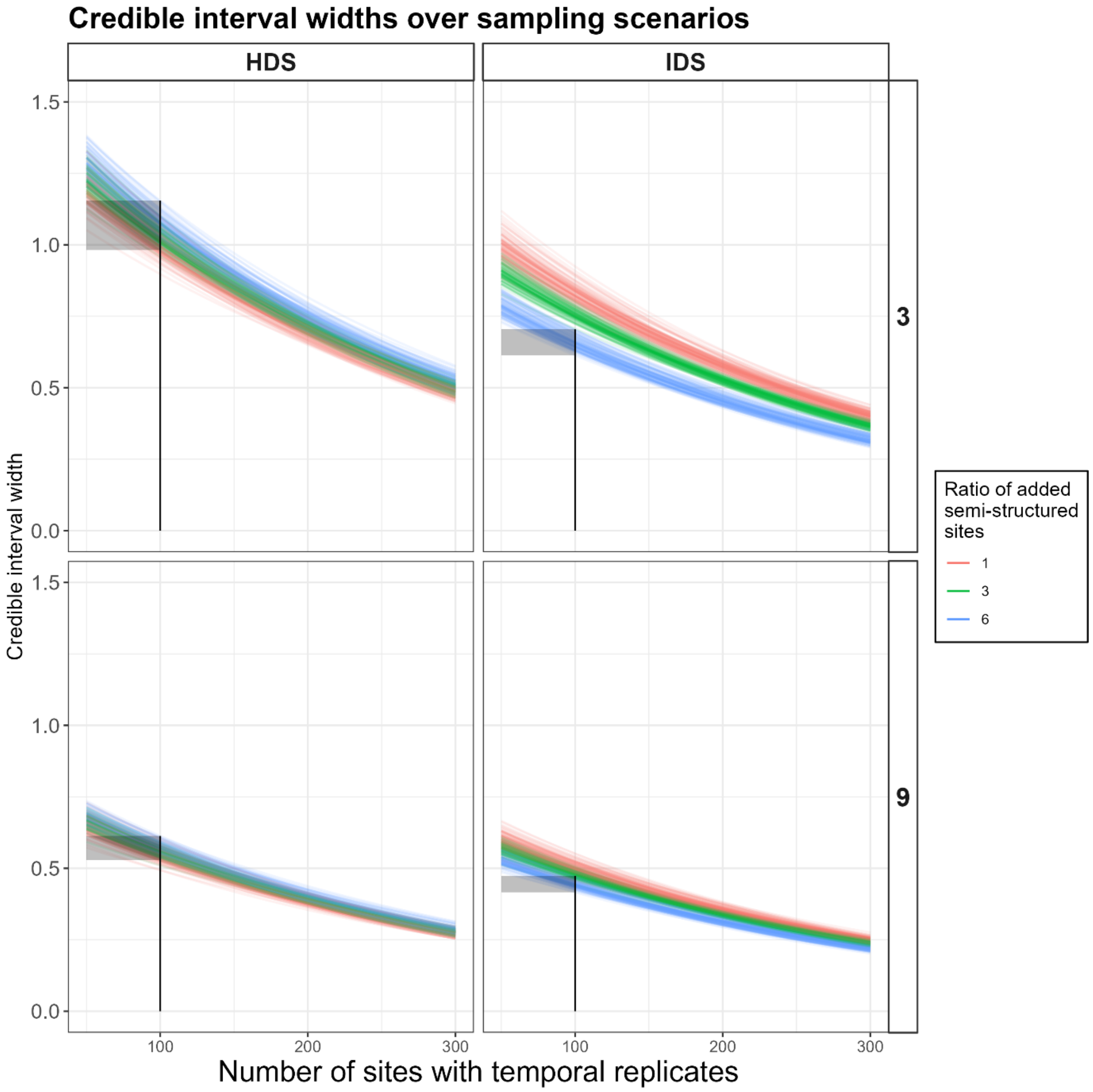

Fig S6.3: CRI average responses and CIs over multiple data collection cases. Ratio of semi-structured data (without temporal replicates) averaged over three classes (1,3 and 6) are depicted by colour-graded lines. HDS and IDS formulation results are depicted by columns, while the number of temporal replicates considered in simulated structured datasets (three or nine) are depicted by row. For visual comparison between the HDS and the IDS estimates accuracy, we plotted CRI responses, grey ribbons, depicting the case of simulated species with high mean detectability (σ_0 = 200m) and high probability of being available (φ_0^{DSopen} = 0.95) surveyed over 100 sites with temporal replicates and six times the number of added semi-structured sites (c), depicted with vertical lines. Lower and upper bounds of the rectangles correspond to minimal and maximal estimated CRI width values.
